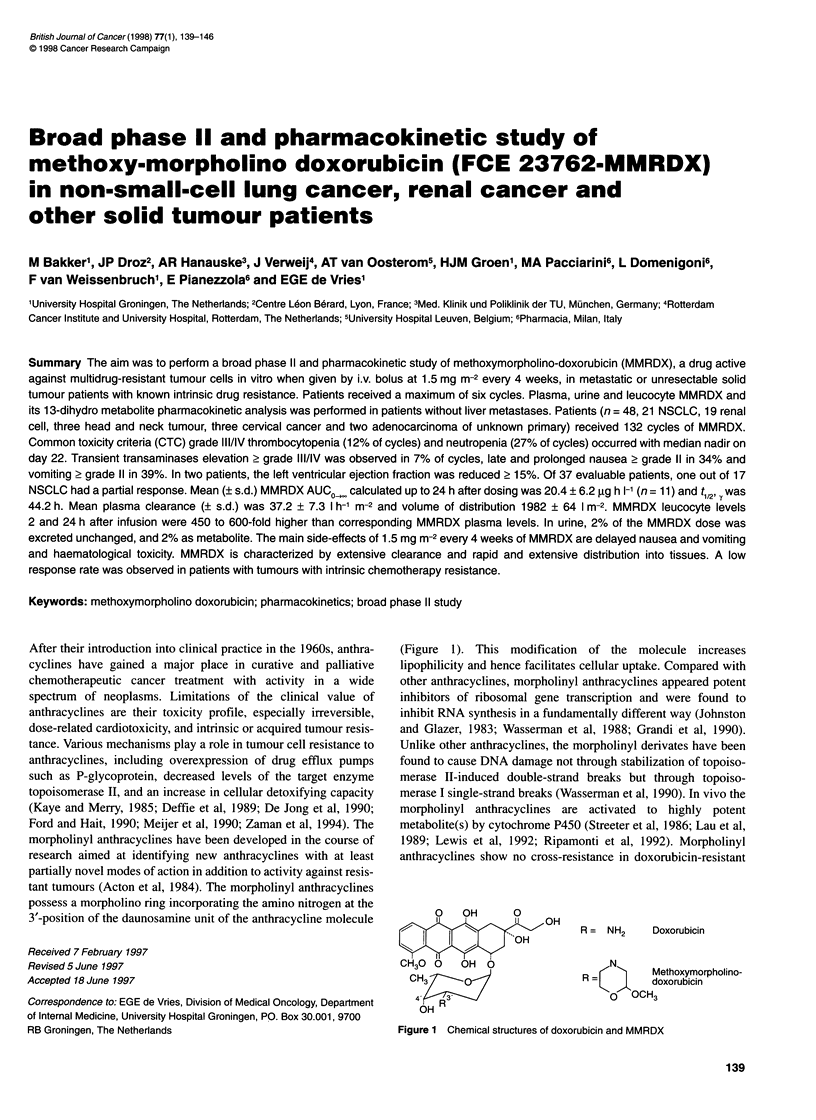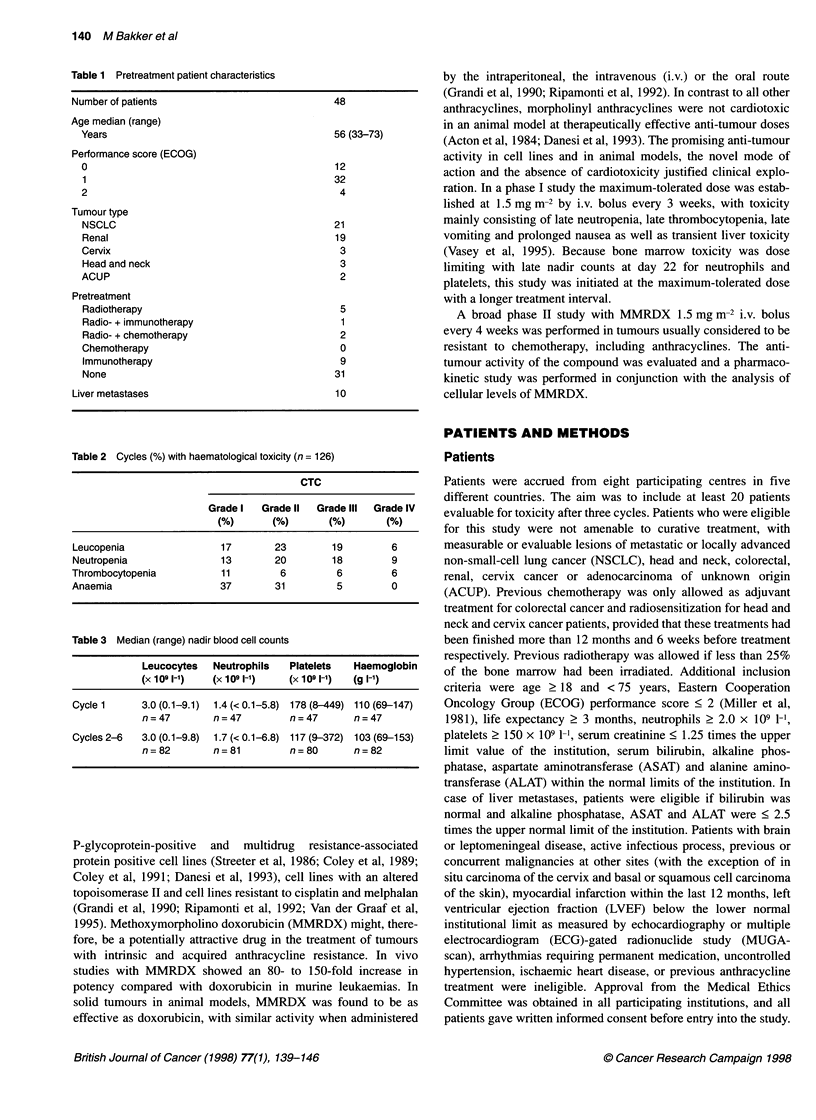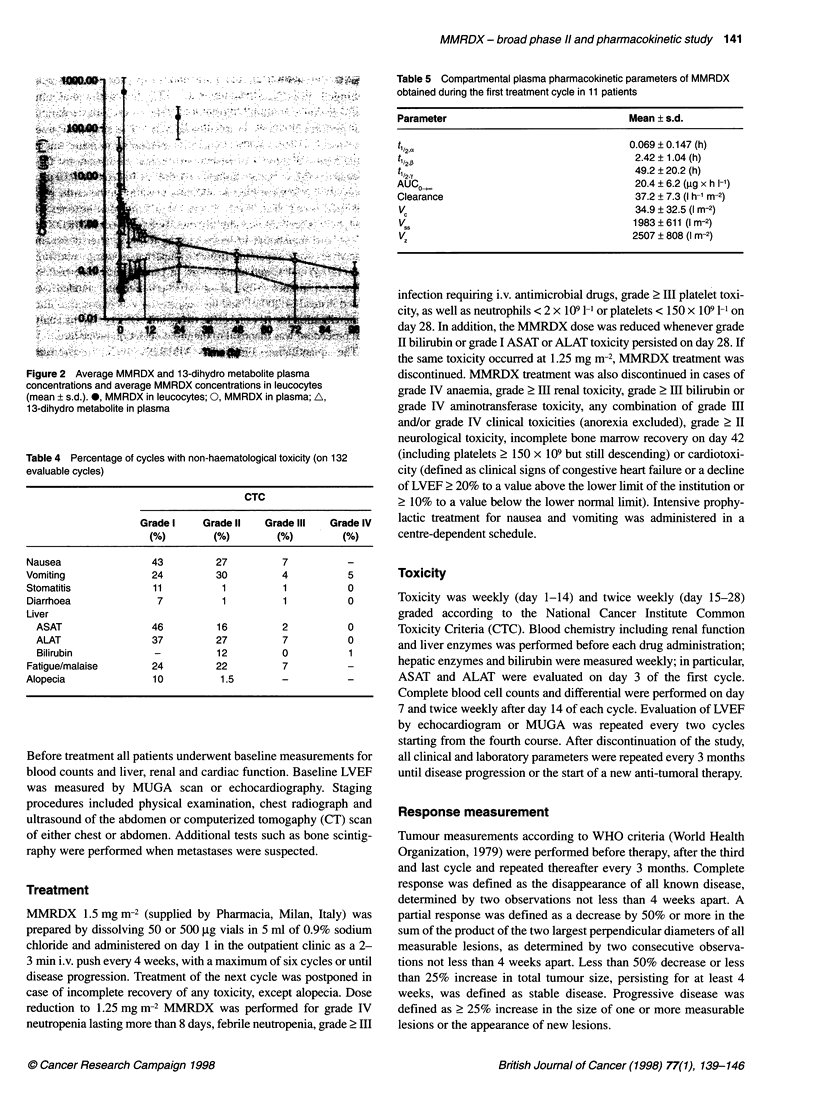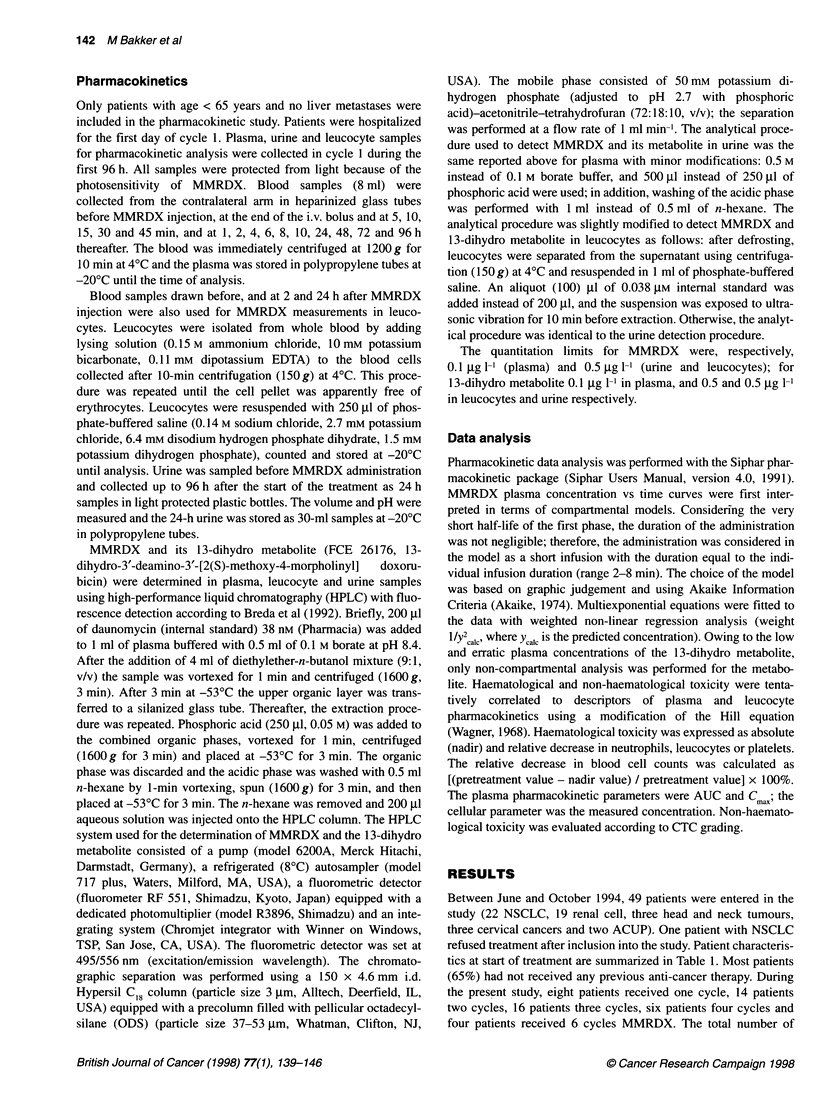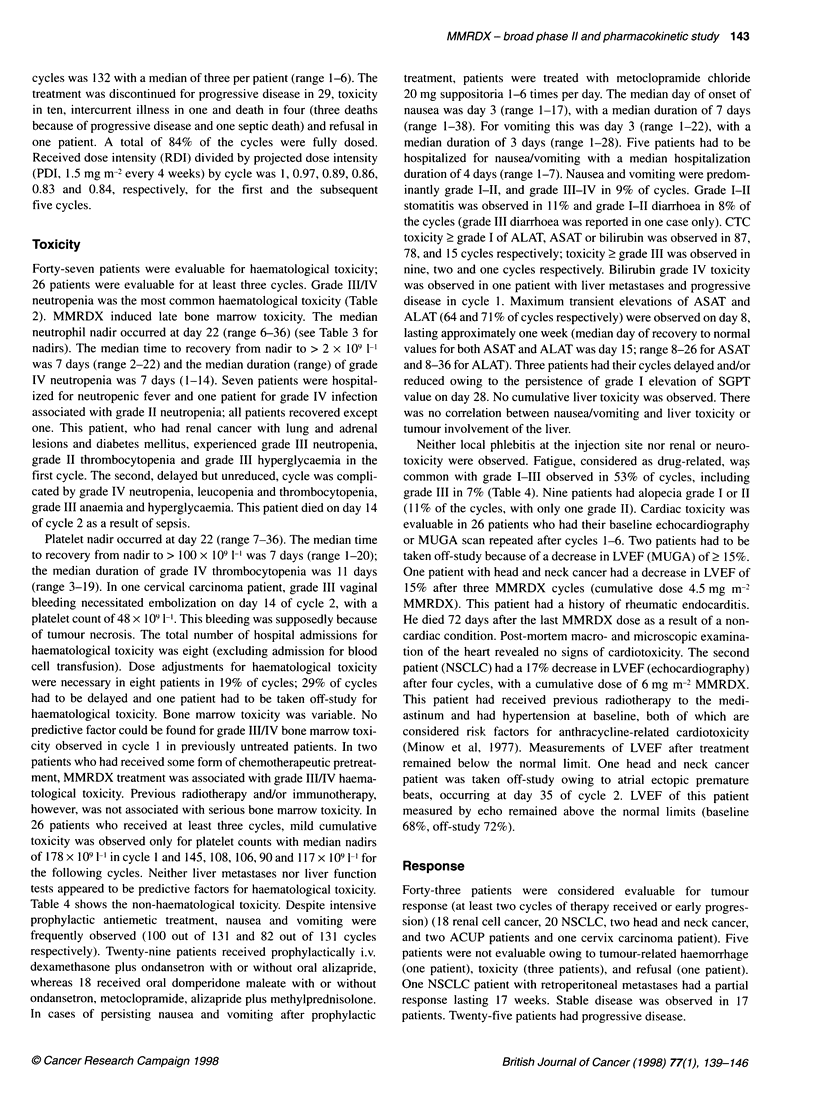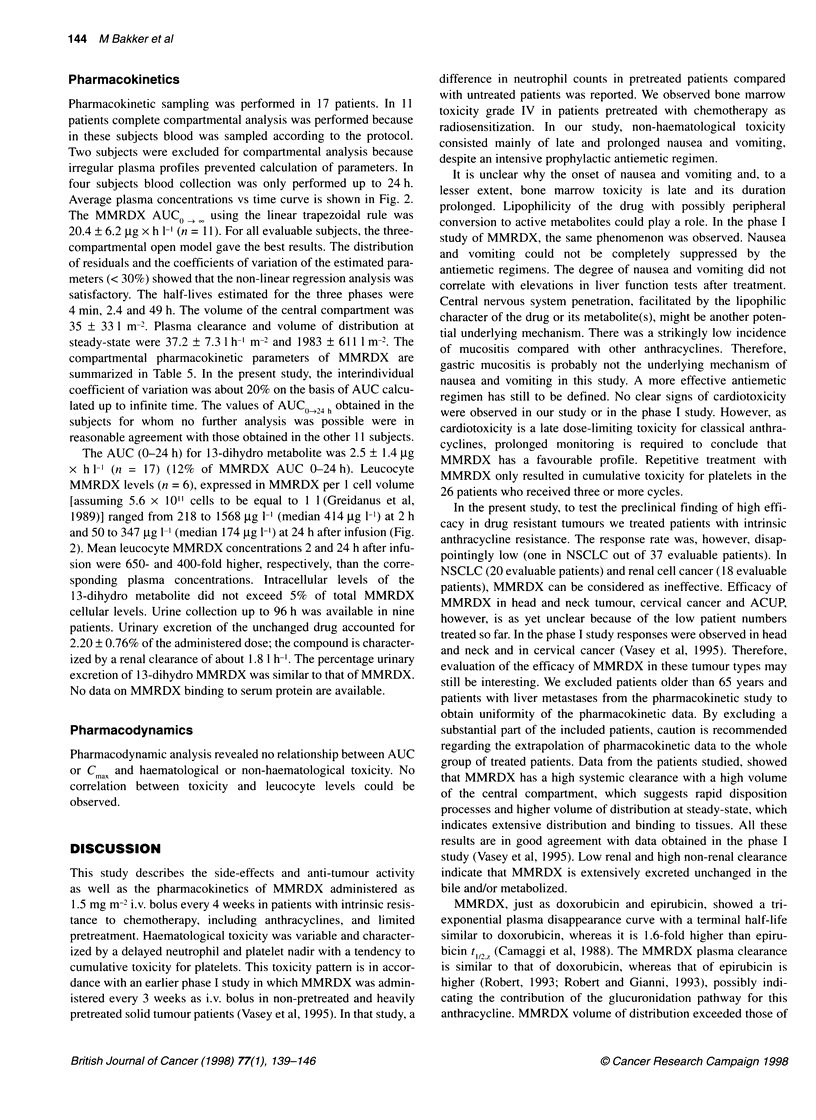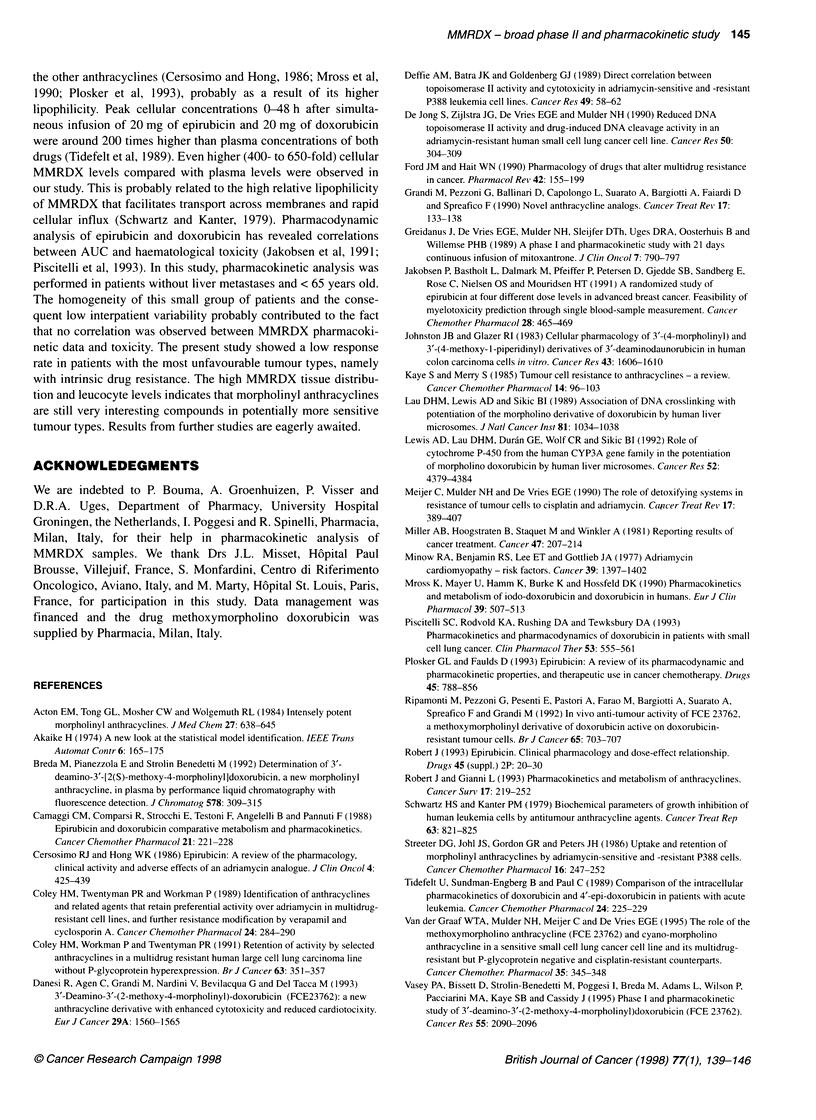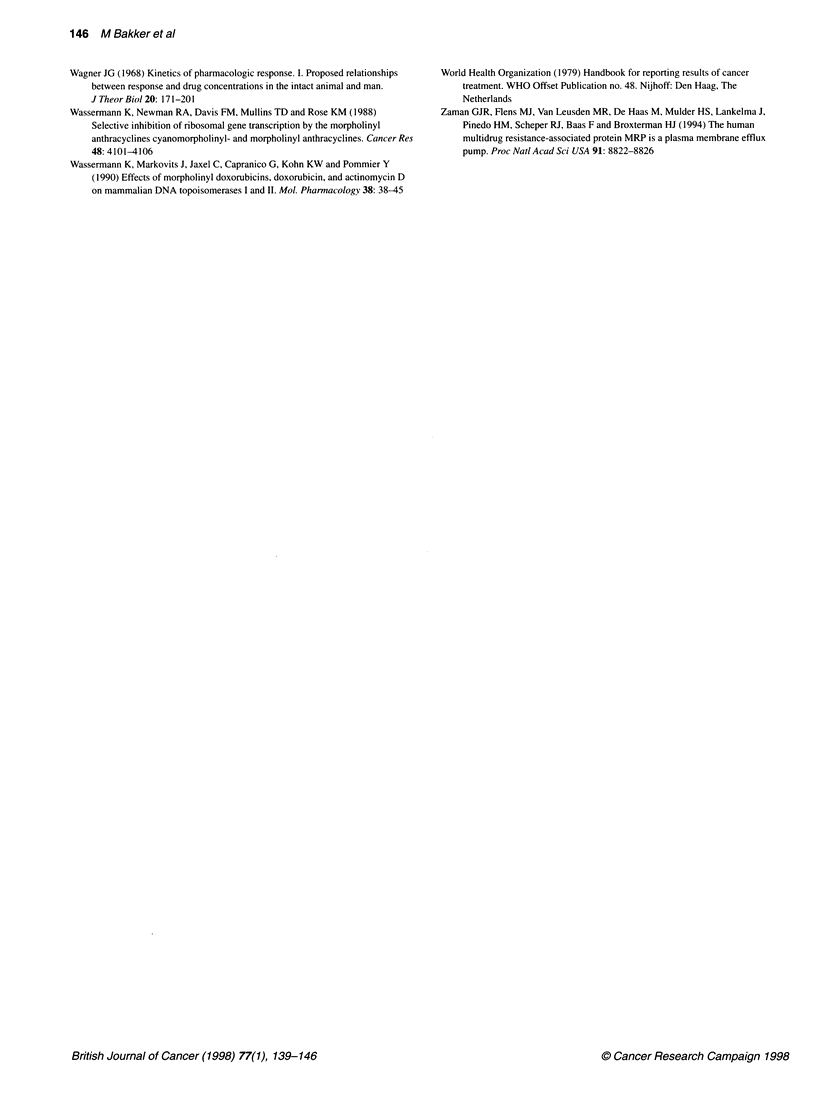# Broad phase II and pharmacokinetic study of methoxy-morpholino doxorubicin (FCE 23762-MMRDX) in non-small-cell lung cancer, renal cancer and other solid tumour patients.

**DOI:** 10.1038/bjc.1998.22

**Published:** 1998

**Authors:** M. Bakker, J. P. Droz, A. R. Hanauske, J. Verweij, A. T. van Oosterom, H. J. Groen, M. A. Pacciarini, L. Domenigoni, F. van Weissenbruch, E. Pianezzola, E. G. de Vries

**Affiliations:** University Hospital Groningen, The Netherlands.

## Abstract

The aim was to perform a broad phase II and pharmacokinetic study of methoxymorpholino-doxorubicin (MMRDX), a drug active against multidrug-resistant tumour cells in vitro when given by i.v. bolus at 1.5 mg m(-2) every 4 weeks, in metastatic or unresectable solid tumour patients with known intrinsic drug resistance. Patients received a maximum of six cycles. Plasma, urine and leucocyte MMRDX and its 13-dihydro metabolite pharmacokinetic analysis was performed in patients without liver metastases. Patients (n = 48, 21 NSCLC, 19 renal cell, three head and neck tumour, three cervical cancer and two adenocarcinoma of unknown primary) received 132 cycles of MMRDX. Common toxicity criteria (CTC) grade III/IV thrombocytopenia (12% of cycles) and neutropenia (27% of cycles) occurred with median nadir on day 22. Transient transaminases elevation > grade III/IV was observed in 7% of cycles, late and prolonged nausea > or = grade II in 34% and vomiting > or = grade II in 39%. In two patients, the left ventricular ejection fraction was reduced > or = 15%. Of 37 evaluable patients, one out of 17 NSCLC had a partial response. Mean (+/- s.d.) MMRDX AUC0-infinity calculated up to 24 h after dosing was 20.4 +/- 6.2 microg h l(-1) (n = 11) and t(1/2, gamma) was 44.2 h. Mean plasma clearance (+/- s.d.) was 37.2 +/- 7.3 l h(-1) m(-2) and volume of distribution 1982 +/- 64 l m(-2). MMRDX leucocyte levels 2 and 24 h after infusion were 450 to 600-fold higher than corresponding MMRDX plasma levels. In urine, 2% of the MMRDX dose was excreted unchanged, and 2% as metabolite. The main side-effects of 1.5 mg m(-2) every 4 weeks of MMRDX are delayed nausea and vomiting and haematological toxicity. MMRDX is characterized by extensive clearance and rapid and extensive distribution into tissues. A low response rate was observed in patients with tumours with intrinsic chemotherapy resistance.


British Journal of Cancer (1998) 77(1), 139–146
© 1998 Cancer Research Campaign

### Broad phase II and pharmacokinetic study of methoxy-morpholino doxorubicin (FCE 23762-MMRDX) in non-small-cell lung cancer, renal cancer and other solid tumour patients

**M Bakker[1], JP Droz[2], AR Hanauske[3], J Verweij[4], AT van Oosterom[5], HJM Groen[1], MA Pacciarini[6], L Domenigoni[6], F van Weissenbruch[1], E Pianezzola[6] and EGE de Vries[1]**

[1]University Hospital Groningen, The Netherlands; [2]Centre Léon Bérard, Lyon, France; [3]Med. Klinik und Poliklinik der TU, München, Germany; [4]Rotterdam Cancer Institute and University Hospital, Rotterdam, The Netherlands; [5]University Hospital Leuven, Belgium; [6]Pharmacia, Milan, Italy

**Summary** The aim was to perform a broad phase II and pharmacokinetic study of methoxymorpholino-doxorubicin (MMRDX), a drug active against multidrug-resistant tumour cells in vitro when given by i.v. bolus at 1.5 mg m$^{-2}$ every 4 weeks, in metastatic or unresectable solid tumour patients with known intrinsic drug resistance. Patients received a maximum of six cycles. Plasma, urine and leucocyte MMRDX and its 13-dihydro metabolite pharmacokinetic analysis was performed in patients without liver metastases. Patients ($n$ = 48, 21 NSCLC, 19 renal cell, three head and neck tumour, three cervical cancer and two adenocarcinoma of unknown primary) received 132 cycles of MMRDX. Common toxicity criteria (CTC) grade III/IV thrombocytopenia (12% of cycles) and neutropenia (27% of cycles) occurred with median nadir on day 22. Transient transaminases elevation ≥ grade III/IV was observed in 7% of cycles, late and prolonged nausea ≥ grade II in 34% and vomiting ≥ grade II in 39%. In two patients, the left ventricular ejection fraction was reduced ≥ 15%. Of 37 evaluable patients, one out of 17 NSCLC had a partial response. Mean (± s.d.) MMRDX $AUC_{0\rightarrow\infty}$ calculated up to 24 h after dosing was 20.4 ± 6.2 μg h l$^{-1}$ ($n$ = 11) and $t_{1/2,\gamma}$ was 44.2 h. Mean plasma clearance (± s.d.) was 37.2 ± 7.3 l h$^{-1}$ m$^{-2}$ and volume of distribution 1982 ± 64 l m$^{-2}$. MMRDX leucocyte levels 2 and 24 h after infusion were 450 to 600-fold higher than corresponding MMRDX plasma levels. In urine, 2% of the MMRDX dose was excreted unchanged, and 2% as metabolite. The main side-effects of 1.5 mg m$^{-2}$ every 4 weeks of MMRDX are delayed nausea and vomiting and haematological toxicity. MMRDX is characterized by extensive clearance and rapid and extensive distribution into tissues. A low response rate was observed in patients with tumours with intrinsic chemotherapy resistance.

**Keywords:** methoxymorpholino doxorubicin; pharmacokinetics; broad phase II study

After their introduction into clinical practice in the 1960s, anthracyclines have gained a major place in curative and palliative chemotherapeutic cancer treatment with activity in a wide spectrum of neoplasms. Limitations of the clinical value of anthracyclines are their toxicity profile, especially irreversible, dose-related cardiotoxicity, and intrinsic or acquired tumour resistance. Various mechanisms play a role in tumour cell resistance to anthracyclines, including overexpression of drug efflux pumps such as P-glycoprotein, decreased levels of the target enzyme topoisomerase II, and an increase in cellular detoxifying capacity (Kaye and Merry, 1985; Deffie et al, 1989; De Jong et al, 1990; Ford and Hait, 1990; Meijer et al, 1990; Zaman et al, 1994). The morpholinyl anthracyclines have been developed in the course of research aimed at identifying new anthracyclines with at least partially novel modes of action in addition to activity against resistant tumours (Acton et al, 1984). The morpholinyl anthracyclines possess a morpholino ring incorporating the amino nitrogen at the 3′-position of the daunosamine unit of the anthracycline molecule (Figure 1). This modification of the molecule increases lipophilicity and hence facilitates cellular uptake. Compared with other anthracyclines, morpholinyl anthracyclines appeared potent inhibitors of ribosomal gene transcription and were found to inhibit RNA synthesis in a fundamentally different way (Johnston and Glazer, 1983; Wasserman et al, 1988; Grandi et al, 1990). Unlike other anthracyclines, the morpholinyl derivates have been found to cause DNA damage not through stabilization of topoisomerase II-induced double-strand breaks but through topoisomerase I single-strand breaks (Wasserman et al, 1990). In vivo the morpholinyl anthracyclines are activated to highly potent metabolite(s) by cytochrome P450 (Streeter et al, 1986; Lau et al, 1989; Lewis et al, 1992; Ripamonti et al, 1992). Morpholinyl anthracyclines show no cross-resistance in doxorubicin-resistant

*Received 7 February 1997*
*Revised 5 June 1997*
*Accepted 18 June 1997*

*Correspondence to:* EGE de Vries, Division of Medical Oncology, Department of Internal Medicine, University Hospital Groningen, P.O. Box 30.001, 9700 RB Groningen, The Netherlands

**Figure 1** Chemical structures of doxorubicin and MMRDX

**Table 1** Pretreatment patient characteristics

| | |
|---|---|
| Number of patients | 48 |
| Age median (range) | |
| Years | 56 (33–73) |
| Performance score (ECOG) | |
| 0 | 12 |
| 1 | 32 |
| 2 | 4 |
| Tumour type | |
| NSCLC | 21 |
| Renal | 19 |
| Cervix | 3 |
| Head and neck | 3 |
| ACUP | 2 |
| Pretreatment | |
| Radiotherapy | 5 |
| Radio- + immunotherapy | 1 |
| Radio- + chemotherapy | 2 |
| Chemotherapy | 0 |
| Immunotherapy | 9 |
| None | 31 |
| Liver metastases | 10 |

**Table 2** Cycles (%) with haematological toxicity ($n$ = 126)

| | CTC | | | |
|---|---|---|---|---|
| | Grade I (%) | Grade II (%) | Grade III (%) | Grade IV (%) |
| Leucopenia | 17 | 23 | 19 | 6 |
| Neutropenia | 13 | 20 | 18 | 9 |
| Thrombocytopenia | 11 | 6 | 6 | 6 |
| Anaemia | 37 | 31 | 5 | 0 |

**Table 3** Median (range) nadir blood cell counts

| | Leucocytes ($\times 10^9$ $l^{-1}$) | Neutrophils ($\times 10^9$ $l^{-1}$) | Platelets ($\times 10^9$ $l^{-1}$) | Haemoglobin (g $l^{-1}$) |
|---|---|---|---|---|
| Cycle 1 | 3.0 (0.1–9.1) $n$ = 47 | 1.4 (< 0.1–5.8) $n$ = 47 | 178 (8–449) $n$ = 47 | 110 (69–147) $n$ = 47 |
| Cycles 2–6 | 3.0 (0.1–9.8) $n$ = 82 | 1.7 (< 0.1–6.8) $n$ = 81 | 117 (9–372) $n$ = 80 | 103 (69–153) $n$ = 82 |

P-glycoprotein-positive and multidrug resistance-associated protein positive cell lines (Streeter et al, 1986; Coley et al, 1989; Coley et al, 1991; Danesi et al, 1993), cell lines with an altered topoisomerase II and cell lines resistant to cisplatin and melphalan (Grandi et al, 1990; Ripamonti et al, 1992; Van der Graaf et al, 1995). Methoxymorpholino doxorubicin (MMRDX) might, therefore, be a potentially attractive drug in the treatment of tumours with intrinsic and acquired anthracycline resistance. In vivo studies with MMRDX showed an 80- to 150-fold increase in potency compared with doxorubicin in murine leukaemias. In solid tumours in animal models, MMRDX was found to be as effective as doxorubicin, with similar activity when administered by the intraperitoneal, the intravenous (i.v.) or the oral route (Grandi et al, 1990; Ripamonti et al, 1992). In contrast to all other anthracyclines, morpholinyl anthracyclines were not cardiotoxic in an animal model at therapeutically effective anti-tumour doses (Acton et al, 1984; Danesi et al, 1993). The promising anti-tumour activity in cell lines and in animal models, the novel mode of action and the absence of cardiotoxicity justified clinical exploration. In a phase I study the maximum-tolerated dose was established at 1.5 mg $m^{-2}$ by i.v. bolus every 3 weeks, with toxicity mainly consisting of late neutropenia, late thrombocytopenia, late vomiting and prolonged nausea as well as transient liver toxicity (Vasey et al, 1995). Because bone marrow toxicity was dose limiting with late nadir counts at day 22 for neutrophils and platelets, this study was initiated at the maximum-tolerated dose with a longer treatment interval.

A broad phase II study with MMRDX 1.5 mg $m^{-2}$ i.v. bolus every 4 weeks was performed in tumours usually considered to be resistant to chemotherapy, including anthracyclines. The anti-tumour activity of the compound was evaluated and a pharmacokinetic study was performed in conjunction with the analysis of cellular levels of MMRDX.

### PATIENTS AND METHODS

### Patients

Patients were accrued from eight participating centres in five different countries. The aim was to include at least 20 patients evaluable for toxicity after three cycles. Patients who were eligible for this study were not amenable to curative treatment, with measurable or evaluable lesions of metastatic or locally advanced non-small-cell lung cancer (NSCLC), head and neck, colorectal, renal, cervix cancer or adenocarcinoma of unknown origin (ACUP). Previous chemotherapy was only allowed as adjuvant treatment for colorectal cancer and radiosensitization for head and neck and cervix cancer patients, provided that these treatments had been finished more than 12 months and 6 weeks before treatment respectively. Previous radiotherapy was allowed if less than 25% of the bone marrow had been irradiated. Additional inclusion criteria were age $\geq 18$ and $< 75$ years, Eastern Cooperation Oncology Group (ECOG) performance score $\leq 2$ (Miller et al, 1981), life expectancy $\geq 3$ months, neutrophils $\geq 2.0 \times 10^9$ $l^{-1}$, platelets $\geq 150 \times 10^9$ $l^{-1}$, serum creatinine $\leq 1.25$ times the upper limit value of the institution, serum bilirubin, alkaline phosphatase, aspartate aminotransferase (ASAT) and alanine aminotransferase (ALAT) within the normal limits of the institution. In case of liver metastases, patients were eligible if bilirubin was normal and alkaline phosphatase, ASAT and ALAT were $\leq 2.5$ times the upper normal limit of the institution. Patients with brain or leptomeningeal disease, active infectious process, previous or concurrent malignancies at other sites (with the exception of in situ carcinoma of the cervix and basal or squamous cell carcinoma of the skin), myocardial infarction within the last 12 months, left ventricular ejection fraction (LVEF) below the lower normal institutional limit as measured by echocardiography or multiple electrocardiogram (ECG)-gated radionuclide study (MUGA-scan), arrhythmias requiring permanent medication, uncontrolled hypertension, ischaemic heart disease, or previous anthracycline treatment were ineligible. Approval from the Medical Ethics Committee was obtained in all participating institutions, and all patients gave written informed consent before entry into the study.

© Cancer Research Campaign 1998

**Figure 2** Average MMRDX and 13-dihydro metabolite plasma concentrations and average MMRDX concentrations in leucocytes (mean ± s.d.). ●, MMRDX in leucocytes; ○, MMRDX in plasma; △, 13-dihydro metabolite in plasma

**Table 4** Percentage of cycles with non-haematological toxicity (on 132 evaluable cycles)

| | CTC | | | |
|---|---|---|---|---|
| | Grade I (%) | Grade II (%) | Grade III (%) | Grade IV (%) |
| Nausea | 43 | 27 | 7 | – |
| Vomiting | 24 | 30 | 4 | 5 |
| Stomatitis | 11 | 1 | 1 | 0 |
| Diarrhoea | 7 | 1 | 1 | 0 |
| Liver | | | | |
| ASAT | 46 | 16 | 2 | 0 |
| ALAT | 37 | 27 | 7 | 0 |
| Bilirubin | – | 12 | 0 | 1 |
| Fatigue/malaise | 24 | 22 | 7 | – |
| Alopecia | 10 | 1.5 | – | – |

Before treatment all patients underwent baseline measurements for blood counts and liver, renal and cardiac function. Baseline LVEF was measured by MUGA scan or echocardiography. Staging procedures included physical examination, chest radiograph and ultrasound of the abdomen or computerized tomogaphy (CT) scan of either chest or abdomen. Additional tests such as bone scintigraphy were performed when metastases were suspected.

### Treatment

MMRDX 1.5 mg $m^{-2}$ (supplied by Pharmacia, Milan, Italy) was prepared by dissolving 50 or 500 µg vials in 5 ml of 0.9% sodium chloride and administered on day 1 in the outpatient clinic as a 2–3 min i.v. push every 4 weeks, with a maximum of six cycles or until disease progression. Treatment of the next cycle was postponed in case of incomplete recovery of any toxicity, except alopecia. Dose reduction to 1.25 mg $m^{-2}$ MMRDX was performed for grade IV neutropenia lasting more than 8 days, febrile neutropenia, grade ≥ III infection requiring i.v. antimicrobial drugs, grade ≥ III platelet toxicity, as well as neutrophils < $2 \times 10^9$ $l^{-1}$ or platelets < $150 \times 10^9$ $l^{-1}$ on day 28. In addition, the MMRDX dose was reduced whenever grade II bilirubin or grade I ASAT or ALAT toxicity persisted on day 28. If the same toxicity occurred at 1.25 mg $m^{-2}$, MMRDX treatment was discontinued. MMRDX treatment was also discontinued in cases of grade IV anaemia, grade ≥ III renal toxicity, grade ≥ III bilirubin or grade IV aminotransferase toxicity, any combination of grade III and/or grade IV clinical toxicities (anorexia excluded), grade ≥ II neurological toxicity, incomplete bone marrow recovery on day 42 (including platelets ≥ $150 \times 10^9$ but still descending) or cardiotoxicity (defined as clinical signs of congestive heart failure or a decline of LVEF ≥ 20% to a value above the lower limit of the institution or ≥ 10% to a value below the lower normal limit). Intensive prophylactic treatment for nausea and vomiting was administered in a centre-dependent schedule.

**Table 5** Compartmental plasma pharmacokinetic parameters of MMRDX obtained during the first treatment cycle in 11 patients

| Parameter | Mean ± s.d. |
|---|---|
| $t_{1/2,\alpha}$ | 0.069 ± 0.147 (h) |
| $t_{1/2,\beta}$ | 2.42 ± 1.04 (h) |
| $t_{1/2,\gamma}$ | 49.2 ± 20.2 (h) |
| $AUC_{0\rightarrow\infty}$ | 20.4 ± 6.2 (µg × h $l^{-1}$) |
| Clearance | 37.2 ± 7.3 (l $h^{-1}$ $m^{-2}$) |
| $V_c$ | 34.9 ± 32.5 (l $m^{-2}$) |
| $V_{ss}$ | 1983 ± 611 (l $m^{-2}$) |
| $V_z$ | 2507 ± 808 (l $m^{-2}$) |

### Toxicity

Toxicity was weekly (day 1–14) and twice weekly (day 15–28) graded according to the National Cancer Institute Common Toxicity Criteria (CTC). Blood chemistry including renal function and liver enzymes was performed before each drug administration; hepatic enzymes and bilirubin were measured weekly; in particular, ASAT and ALAT were evaluated on day 3 of the first cycle. Complete blood cell counts and differential were performed on day 7 and twice weekly after day 14 of each cycle. Evaluation of LVEF by echocardiogram or MUGA was repeated every two cycles starting from the fourth course. After discontinuation of the study, all clinical and laboratory parameters were repeated every 3 months until disease progression or the start of a new anti-tumoral therapy.

### Response measurement

Tumour measurements according to WHO criteria (World Health Organization, 1979) were performed before therapy, after the third and last cycle and repeated thereafter every 3 months. Complete response was defined as the disappearance of all known disease, determined by two observations not less than 4 weeks apart. A partial response was defined as a decrease by 50% or more in the sum of the product of the two largest perpendicular diameters of all measurable lesions, as determined by two consecutive observations not less than 4 weeks apart. Less than 50% decrease or less than 25% increase in total tumour size, persisting for at least 4 weeks, was defined as stable disease. Progressive disease was defined as ≥ 25% increase in the size of one or more measurable lesions or the appearance of new lesions.

© Cancer Research Campaign 1998

### Pharmacokinetics

Only patients with age < 65 years and no liver metastases were included in the pharmacokinetic study. Patients were hospitalized for the first day of cycle 1. Plasma, urine and leucocyte samples for pharmacokinetic analysis were collected in cycle 1 during the first 96 h. All samples were protected from light because of the photosensitivity of MMRDX. Blood samples (8 ml) were collected from the contralateral arm in heparinized glass tubes before MMRDX injection, at the end of the i.v. bolus and at 5, 10, 15, 30 and 45 min, and at 1, 2, 4, 6, 8, 10, 24, 48, 72 and 96 h thereafter. The blood was immediately centrifuged at 1200 **g** for 10 min at 4°C and the plasma was stored in polypropylene tubes at –20°C until the time of analysis.

Blood samples drawn before, and at 2 and 24 h after MMRDX injection were also used for MMRDX measurements in leucocytes. Leucocytes were isolated from whole blood by adding lysing solution (0.15 M ammonium chloride, 10 mM potassium bicarbonate, 0.11 mM dipotassium EDTA) to the blood cells collected after 10-min centrifugation (150 **g**) at 4°C. This procedure was repeated until the cell pellet was apparently free of erythrocytes. Leucocytes were resuspended with 250 µl of phosphate-buffered saline (0.14 M sodium chloride, 2.7 mM potassium chloride, 6.4 mM disodium hydrogen phosphate dihydrate, 1.5 mM potassium dihydrogen phosphate), counted and stored at –20°C until analysis. Urine was sampled before MMRDX administration and collected up to 96 h after the start of the treatment as 24 h samples in light protected plastic bottles. The volume and pH were measured and the 24-h urine was stored as 30-ml samples at –20°C in polypropylene tubes.

MMRDX and its 13-dihydro metabolite (FCE 26176, 13-dihydro-3′-deamino-3′-[2(S)-methoxy-4-morpholinyl] doxorubicin) were determined in plasma, leucocyte and urine samples using high-performance liquid chromatography (HPLC) with fluorescence detection according to Breda et al (1992). Briefly, 200 µl of daunomycin (internal standard) 38 nM (Pharmacia) was added to 1 ml of plasma buffered with 0.5 ml of 0.1 M borate at pH 8.4. After the addition of 4 ml of diethylether-*n*-butanol mixture (9:1, v/v) the sample was vortexed for 1 min and centrifuged (1600 **g**, 3 min). After 3 min at –53°C the upper organic layer was transferred to a silanized glass tube. Thereafter, the extraction procedure was repeated. Phosphoric acid (250 µl, 0.05 M) was added to the combined organic phases, vortexed for 1 min, centrifuged (1600 **g** for 3 min) and placed at –53°C for 3 min. The organic phase was discarded and the acidic phase was washed with 0.5 ml *n*-hexane by 1-min vortexing, spun (1600 **g**) for 3 min, and then placed at –53°C for 3 min. The *n*-hexane was removed and 200 µl aqueous solution was injected onto the HPLC column. The HPLC system used for the determination of MMRDX and the 13-dihydro metabolite consisted of a pump (model 6200A, Merck Hitachi, Darmstadt, Germany), a refrigerated (8°C) autosampler (model 717 plus, Waters, Milford, MA, USA), a fluorometric detector (fluorometer RF 551, Shimadzu, Kyoto, Japan) equipped with a dedicated photomultiplier (model R3896, Shimadzu) and an integrating system (Chromjet integrator with Winner on Windows, TSP, San Jose, CA, USA). The fluorometric detector was set at 495/556 nm (excitation/emission wavelength). The chromatographic separation was performed using a 150 × 4.6 mm i.d. Hypersil $C_{18}$ column (particle size 3 µm, Alltech, Deerfield, IL, USA) equipped with a precolumn filled with pellicular octadecylsilane (ODS) (particle size 37–53 µm, Whatman, Clifton, NJ, USA). The mobile phase consisted of 50 mM potassium dihydrogen phosphate (adjusted to pH 2.7 with phosphoric acid)–acetonitrile–tetrahydrofuran (72:18:10, v/v); the separation was performed at a flow rate of 1 ml $min^{-1}$. The analytical procedure used to detect MMRDX and its metabolite in urine was the same reported above for plasma with minor modifications: 0.5 M instead of 0.1 M borate buffer, and 500 µl instead of 250 µl of phosphoric acid were used; in addition, washing of the acidic phase was performed with 1 ml instead of 0.5 ml of *n*-hexane. The analytical procedure was slightly modified to detect MMRDX and 13-dihydro metabolite in leucocytes as follows: after defrosting, leucocytes were separated from the supernatant using centrifugation (150 **g**) at 4°C and resuspended in 1 ml of phosphate-buffered saline. An aliquot (100) µl of 0.038 µM internal standard was added instead of 200 µl, and the suspension was exposed to ultrasonic vibration for 10 min before extraction. Otherwise, the analytical procedure was identical to the urine detection procedure.

The quantitation limits for MMRDX were, respectively, 0.1 µg $l^{-1}$ (plasma) and 0.5 µg $l^{-1}$ (urine and leucocytes); for 13-dihydro metabolite 0.1 µg $l^{-1}$ in plasma, and 0.5 and 0.5 µg $l^{-1}$ in leucocytes and urine respectively.

### Data analysis

Pharmacokinetic data analysis was performed with the Siphar pharmacokinetic package (Siphar Users Manual, version 4.0, 1991). MMRDX plasma concentration vs time curves were first interpreted in terms of compartmental models. Considering the very short half-life of the first phase, the duration of the administration was not negligible; therefore, the administration was considered in the model as a short infusion with the duration equal to the individual infusion duration (range 2–8 min). The choice of the model was based on graphic judgement and using Akaike Information Criteria (Akaike, 1974). Multiexponential equations were fitted to the data with weighted non-linear regression analysis (weight $1/y^2_{calc}$, where $y_{calc}$ is the predicted concentration). Owing to the low and erratic plasma concentrations of the 13-dihydro metabolite, only non-compartmental analysis was performed for the metabolite. Haematological and non-haematological toxicity were tentatively correlated to descriptors of plasma and leucocyte pharmacokinetics using a modification of the Hill equation (Wagner, 1968). Haematological toxicity was expressed as absolute (nadir) and relative decrease in neutrophils, leucocytes or platelets. The relative decrease in blood cell counts was calculated as [(pretreatment value – nadir value) / pretreatment value] × 100%. The plasma pharmacokinetic parameters were AUC and $C_{max}$; the cellular parameter was the measured concentration. Non-haematological toxicity was evaluated according to CTC grading.

### RESULTS

Between June and October 1994, 49 patients were entered in the study (22 NSCLC, 19 renal cell, three head and neck tumours, three cervical cancers and two ACUP). One patient with NSCLC refused treatment after inclusion into the study. Patient characteristics at start of treatment are summarized in Table 1. Most patients (65%) had not received any previous anti-cancer therapy. During the present study, eight patients received one cycle, 14 patients two cycles, 16 patients three cycles, six patients four cycles and four patients received 6 cycles MMRDX. The total number of

 © Cancer Research Campaign 1998

cycles was 132 with a median of three per patient (range 1–6). The treatment was discontinued for progressive disease in 29, toxicity in ten, intercurrent illness in one and death in four (three deaths because of progressive disease and one septic death) and refusal in one patient. A total of 84% of the cycles were fully dosed. Received dose intensity (RDI) divided by projected dose intensity (PDI, 1.5 mg $m^{-2}$ every 4 weeks) by cycle was 1, 0.97, 0.89, 0.86, 0.83 and 0.84, respectively, for the first and the subsequent five cycles.

### Toxicity

Forty-seven patients were evaluable for haematological toxicity; 26 patients were evaluable for at least three cycles. Grade III/IV neutropenia was the most common haematological toxicity (Table 2). MMRDX induced late bone marrow toxicity. The median neutrophil nadir occurred at day 22 (range 6–36) (see Table 3 for nadirs). The median time to recovery from nadir to > 2 × $10^9$ $l^{-1}$ was 7 days (range 2–22) and the median duration (range) of grade IV neutropenia was 7 days (1–14). Seven patients were hospitalized for neutropenic fever and one patient for grade IV infection associated with grade II neutropenia; all patients recovered except one. This patient, who had renal cancer with lung and adrenal lesions and diabetes mellitus, experienced grade III neutropenia, grade II thrombocytopenia and grade III hyperglycaemia in the first cycle. The second, delayed but unreduced, cycle was complicated by grade IV neutropenia, leucopenia and thrombocytopenia, grade III anaemia and hyperglycaemia. This patient died on day 14 of cycle 2 as a result of sepsis.

Platelet nadir occurred at day 22 (range 7–36). The median time to recovery from nadir to > 100 × $10^9$ $l^{-1}$ was 7 days (range 1–20); the median duration of grade IV thrombocytopenia was 11 days (range 3–19). In one cervical carcinoma patient, grade III vaginal bleeding necessitated embolization on day 14 of cycle 2, with a platelet count of 48 × $10^9$ $l^{-1}$. This bleeding was supposedly because of tumour necrosis. The total number of hospital admissions for haematological toxicity was eight (excluding admission for blood cell transfusion). Dose adjustments for haematological toxicity were necessary in eight patients in 19% of cycles; 29% of cycles had to be delayed and one patient had to be taken off-study for haematological toxicity. Bone marrow toxicity was variable. No predictive factor could be found for grade III/IV bone marrow toxicity observed in cycle 1 in previously untreated patients. In two patients who had received some form of chemotherapeutic pretreatment, MMRDX treatment was associated with grade III/IV haematological toxicity. Previous radiotherapy and/or immunotherapy, however, was not associated with serious bone marrow toxicity. In 26 patients who received at least three cycles, mild cumulative toxicity was observed only for platelet counts with median nadirs of 178 × $10^9$ $l^{-1}$ in cycle 1 and 145, 108, 106, 90 and 117 × $10^9$ $l^{-1}$ for the following cycles. Neither liver metastases nor liver function tests appeared to be predictive factors for haematological toxicity. Table 4 shows the non-haematological toxicity. Despite intensive prophylactic antiemetic treatment, nausea and vomiting were frequently observed (100 out of 131 and 82 out of 131 cycles respectively). Twenty-nine patients received prophylactically i.v. dexamethasone plus ondansetron with or without oral alizapride, whereas 18 received oral domperidone maleate with or without ondansetron, metoclopramide, alizapride plus methylprednisolone. In cases of persisting nausea and vomiting after prophylactic treatment, patients were treated with metoclopramide chloride 20 mg suppositoria 1–6 times per day. The median day of onset of nausea was day 3 (range 1–17), with a median duration of 7 days (range 1–38). For vomiting this was day 3 (range 1–22), with a median duration of 3 days (range 1–28). Five patients had to be hospitalized for nausea/vomiting with a median hospitalization duration of 4 days (range 1–7). Nausea and vomiting were predominantly grade I–II, and grade III–IV in 9% of cycles. Grade I–II stomatitis was observed in 11% and grade I–II diarrhoea in 8% of the cycles (grade III diarrhoea was reported in one case only). CTC toxicity ≥ grade I of ALAT, ASAT or bilirubin was observed in 87, 78, and 15 cycles respectively; toxicity ≥ grade III was observed in nine, two and one cycles respectively. Bilirubin grade IV toxicity was observed in one patient with liver metastases and progressive disease in cycle 1. Maximum transient elevations of ASAT and ALAT (64 and 71% of cycles respectively) were observed on day 8, lasting approximately one week (median day of recovery to normal values for both ASAT and ALAT was day 15; range 8–26 for ASAT and 8–36 for ALAT). Three patients had their cycles delayed and/or reduced owing to the persistence of grade I elevation of SGPT value on day 28. No cumulative liver toxicity was observed. There was no correlation between nausea/vomiting and liver toxicity or tumour involvement of the liver.

Neither local phlebitis at the injection site nor renal or neurotoxicity were observed. Fatigue, considered as drug-related, was common with grade I–III observed in 53% of cycles, including grade III in 7% (Table 4). Nine patients had alopecia grade I or II (11% of the cycles, with only one grade II). Cardiac toxicity was evaluable in 26 patients who had their baseline echocardiography or MUGA scan repeated after cycles 1–6. Two patients had to be taken off-study because of a decrease in LVEF (MUGA) of ≥ 15%. One patient with head and neck cancer had a decrease in LVEF of 15% after three MMRDX cycles (cumulative dose 4.5 mg $m^{-2}$ MMRDX). This patient had a history of rheumatic endocarditis. He died 72 days after the last MMRDX dose as a result of a non-cardiac condition. Post-mortem macro- and microscopic examination of the heart revealed no signs of cardiotoxicity. The second patient (NSCLC) had a 17% decrease in LVEF (echocardiography) after four cycles, with a cumulative dose of 6 mg $m^{-2}$ MMRDX. This patient had received previous radiotherapy to the mediastinum and had hypertension at baseline, both of which are considered risk factors for anthracycline-related cardiotoxicity (Minow et al, 1977). Measurements of LVEF after treatment remained below the normal limit. One head and neck cancer patient was taken off-study owing to atrial ectopic premature beats, occurring at day 35 of cycle 2. LVEF of this patient measured by echo remained above the normal limits (baseline 68%, off-study 72%).

### Response

Forty-three patients were considered evaluable for tumour response (at least two cycles of therapy received or early progression) (18 renal cell cancer, 20 NSCLC, two head and neck cancer, and two ACUP patients and one cervix carcinoma patient). Five patients were not evaluable owing to tumour-related haemorrhage (one patient), toxicity (three patients), and refusal (one patient). One NSCLC patient with retroperitoneal metastases had a partial response lasting 17 weeks. Stable disease was observed in 17 patients. Twenty-five patients had progressive disease.

© Cancer Research Campaign 1998

### Pharmacokinetics

Pharmacokinetic sampling was performed in 17 patients. In 11 patients complete compartmental analysis was performed because in these subjects blood was sampled according to the protocol. Two subjects were excluded for compartmental analysis because irregular plasma profiles prevented calculation of parameters. In four subjects blood collection was only performed up to 24 h. Average plasma concentrations vs time curve is shown in Fig. 2. The MMRDX $AUC_{0 \rightarrow \infty}$ using the linear trapezoidal rule was $20.4 \pm 6.2$ μg × h $l^{-1}$ ($n = 11$). For all evaluable subjects, the three-compartmental open model gave the best results. The distribution of residuals and the coefficients of variation of the estimated parameters (< 30%) showed that the non-linear regression analysis was satisfactory. The half-lives estimated for the three phases were 4 min, 2.4 and 49 h. The volume of the central compartment was 35 ± 33 l $m^{-2}$. Plasma clearance and volume of distribution at steady-state were 37.2 ± 7.3 l $h^{-1}$ $m^{-2}$ and 1983 ± 611 l $m^{-2}$. The compartmental pharmacokinetic parameters of MMRDX are summarized in Table 5. In the present study, the interindividual coefficient of variation was about 20% on the basis of AUC calculated up to infinite time. The values of $AUC_{0 \rightarrow 24\ h}$ obtained in the subjects for whom no further analysis was possible were in reasonable agreement with those obtained in the other 11 subjects.

The AUC (0–24 h) for 13-dihydro metabolite was 2.5 ± 1.4 μg × h $l^{-1}$ ($n = 17$) (12% of MMRDX AUC 0–24 h). Leucocyte MMRDX levels ($n = 6$), expressed in MMRDX per 1 cell volume [assuming $5.6 \times 10^{11}$ cells to be equal to 1 l (Greidanus et al, 1989)] ranged from 218 to 1568 μg $l^{-1}$ (median 414 μg $l^{-1}$) at 2 h and 50 to 347 μg $l^{-1}$ (median 174 μg $l^{-1}$) at 24 h after infusion (Fig. 2). Mean leucocyte MMRDX concentrations 2 and 24 h after infusion were 650- and 400-fold higher, respectively, than the corresponding plasma concentrations. Intracellular levels of the 13-dihydro metabolite did not exceed 5% of total MMRDX cellular levels. Urine collection up to 96 h was available in nine patients. Urinary excretion of the unchanged drug accounted for 2.20 ± 0.76% of the administered dose; the compound is characterized by a renal clearance of about 1.8 l $h^{-1}$. The percentage urinary excretion of 13-dihydro MMRDX was similar to that of MMRDX. No data on MMRDX binding to serum protein are available.

### Pharmacodynamics

Pharmacodynamic analysis revealed no relationship between AUC or $C_{max}$ and haematological or non-haematological toxicity. No correlation between toxicity and leucocyte levels could be observed.

### DISCUSSION

This study describes the side-effects and anti-tumour activity as well as the pharmacokinetics of MMRDX administered as 1.5 mg $m^{-2}$ i.v. bolus every 4 weeks in patients with intrinsic resistance to chemotherapy, including anthracyclines, and limited pretreatment. Haematological toxicity was variable and characterized by a delayed neutrophil and platelet nadir with a tendency to cumulative toxicity for platelets. This toxicity pattern is in accordance with an earlier phase I study in which MMRDX was administered every 3 weeks as i.v. bolus in non-pretreated and heavily pretreated solid tumour patients (Vasey et al, 1995). In that study, a difference in neutrophil counts in pretreated patients compared with untreated patients was reported. We observed bone marrow toxicity grade IV in patients pretreated with chemotherapy as radiosensitization. In our study, non-haematological toxicity consisted mainly of late and prolonged nausea and vomiting, despite an intensive prophylactic antiemetic regimen.

It is unclear why the onset of nausea and vomiting and, to a lesser extent, bone marrow toxicity is late and its duration prolonged. Lipophilicity of the drug with possibly peripheral conversion to active metabolites could play a role. In the phase I study of MMRDX, the same phenomenon was observed. Nausea and vomiting could not be completely suppressed by the antiemetic regimens. The degree of nausea and vomiting did not correlate with elevations in liver function tests after treatment. Central nervous system penetration, facilitated by the lipophilic character of the drug or its metabolite(s), might be another potential underlying mechanism. There was a strikingly low incidence of mucositis compared with other anthracyclines. Therefore, gastric mucositis is probably not the underlying mechanism of nausea and vomiting in this study. A more effective antiemetic regimen has still to be defined. No clear signs of cardiotoxicity were observed in our study or in the phase I study. However, as cardiotoxicity is a late dose-limiting toxicity for classical anthracyclines, prolonged monitoring is required to conclude that MMRDX has a favourable profile. Repetitive treatment with MMRDX only resulted in cumulative toxicity for platelets in the 26 patients who received three or more cycles.

In the present study, to test the preclinical finding of high efficacy in drug resistant tumours we treated patients with intrinsic anthracycline resistance. The response rate was, however, disappointingly low (one in NSCLC out of 37 evaluable patients). In NSCLC (20 evaluable patients) and renal cell cancer (18 evaluable patients), MMRDX can be considered as ineffective. Efficacy of MMRDX in head and neck tumour, cervical cancer and ACUP, however, is as yet unclear because of the low patient numbers treated so far. In the phase I study responses were observed in head and neck and in cervical cancer (Vasey et al, 1995). Therefore, evaluation of the efficacy of MMRDX in these tumour types may still be interesting. We excluded patients older than 65 years and patients with liver metastases from the pharmacokinetic study to obtain uniformity of the pharmacokinetic data. By excluding a substantial part of the included patients, caution is recommended regarding the extrapolation of pharmacokinetic data to the whole group of treated patients. Data from the patients studied, showed that MMRDX has a high systemic clearance with a high volume of the central compartment, which suggests rapid disposition processes and higher volume of distribution at steady-state, which indicates extensive distribution and binding to tissues. All these results are in good agreement with data obtained in the phase I study (Vasey et al, 1995). Low renal and high non-renal clearance indicate that MMRDX is extensively excreted unchanged in the bile and/or metabolized.

MMRDX, just as doxorubicin and epirubicin, showed a tri-exponential plasma disappearance curve with a terminal half-life similar to doxorubicin, whereas it is 1.6-fold higher than epirubicin $t_{1/2,z}$ (Camaggi et al, 1988). The MMRDX plasma clearance is similar to that of doxorubicin, whereas that of epirubicin is higher (Robert, 1993; Robert and Gianni, 1993), possibly indicating the contribution of the glucuronidation pathway for this anthracycline. MMRDX volume of distribution exceeded those of

© Cancer Research Campaign 1998

the other anthracyclines (Cersosimo and Hong, 1986; Mross et al, 1990; Plosker et al, 1993), probably as a result of its higher lipophilicity. Peak cellular concentrations 0–48 h after simultaneous infusion of 20 mg of epirubicin and 20 mg of doxorubicin were around 200 times higher than plasma concentrations of both drugs (Tidefelt et al, 1989). Even higher (400- to 650-fold) cellular MMRDX levels compared with plasma levels were observed in our study. This is probably related to the high relative lipophilicity of MMRDX that facilitates transport across membranes and rapid cellular influx (Schwartz and Kanter, 1979). Pharmacodynamic analysis of epirubicin and doxorubicin has revealed correlations between AUC and haematological toxicity (Jakobsen et al, 1991; Piscitelli et al, 1993). In this study, pharmacokinetic analysis was performed in patients without liver metastases and < 65 years old. The homogeneity of this small group of patients and the consequent low interpatient variability probably contributed to the fact that no correlation was observed between MMRDX pharmacokinetic data and toxicity. The present study showed a low response rate in patients with the most unfavourable tumour types, namely with intrinsic drug resistance. The high MMRDX tissue distribution and leucocyte levels indicates that morpholinyl anthracyclines are still very interesting compounds in potentially more sensitive tumour types. Results from further studies are eagerly awaited.

### ACKNOWLEDGEMENTS

We are indebted to P. Bouma, A. Groenhuizen, P. Visser and D.R.A. Uges, Department of Pharmacy, University Hospital Groningen, the Netherlands, I. Poggesi and R. Spinelli, Pharmacia, Milan, Italy, for their help in pharmacokinetic analysis of MMRDX samples. We thank Drs J.L. Misset, Hôpital Paul Brousse, Villejuif, France, S. Monfardini, Centro di Riferimento Oncologico, Aviano, Italy, and M. Marty, Hôpital St. Louis, Paris, France, for participation in this study. Data management was financed and the drug methoxymorpholino doxorubicin was supplied by Pharmacia, Milan, Italy.

### REFERENCES

Acton EM, Tong GL, Mosher CW and Wolgemuth RL (1984) Intensely potent morpholinyl anthracyclines. *J Med Chem* **27**: 638–645

Akaike H (1974) A new look at the statistical model identification. *IEEE Trans Automat Contr* **6**: 165–175

Breda M, Pianezzola E and Strolin Benedetti M (1992) Determination of 3′-deamino-3′-[2(S)-methoxy-4-morpholinyl]doxorubicin, a new morpholinyl anthracycline, in plasma by performance liquid chromatography with fluorescence detection. *J Chromatog* **578**: 309–315

Camaggi CM, Comparsi R, Strocchi E, Testoni F, Angelelli B and Pannuti F (1988) Epirubicin and doxorubicin comparative metabolism and pharmacokinetics. *Cancer Chemother Pharmacol* **21**: 221–228

Cersosimo RJ and Hong WK (1986) Epirubicin: A review of the pharmacology, clinical activity and adverse effects of an adriamycin analogue. *J Clin Oncol* **4**: 425–439

Coley HM, Twentyman PR and Workman P (1989) Identification of anthracyclines and related agents that retain preferential activity over adriamycin in multidrug-resistant cell lines, and further resistance modification by verapamil and cyclosporin A. *Cancer Chemother Pharmacol* **24**: 284–290

Coley HM, Workman P and Twentyman PR (1991) Retention of activity by selected anthracyclines in a multidrug resistant human large cell lung carcinoma line without P-glycoprotein hyperexpression. *Br J Cancer* **63**: 351–357

Danesi R, Agen C, Grandi M, Nardini V, Bevilacqua G and Del Tacca M (1993) 3′-Deamino-3′-(2-methoxy-4-morpholinyl)-doxorubicin (FCE23762): a new anthracycline derivative with enhanced cytotoxicity and reduced cardiotocixity. *Eur J Cancer* **29A**: 1560–1565

Deffie AM, Batra JK and Goldenberg GJ (1989) Direct correlation between topoisomerase II activity and cytotoxicity in adriamycin-sensitive and -resistant P388 leukemia cell lines. *Cancer Res* **49**: 58–62

De Jong S, Zijlstra JG, De Vries EGE and Mulder NH (1990) Reduced DNA topoisomerase II activity and drug-induced DNA cleavage activity in an adriamycin-resistant human small cell lung cancer cell line. *Cancer Res* **50**: 304–309

Ford JM and Hait WN (1990) Pharmacology of drugs that alter multidrug resistance in cancer. *Pharmacol Rev* **42**: 155–199

Grandi M, Pezzoni G, Ballinari D, Capolongo L, Suarato A, Bargiotti A, Faiardi D and Spreafico F (1990) Novel anthracycline analogs. *Cancer Treat Rev* **17**: 133–138

Greidanus J, De Vries EGE, Mulder NH, Sleijfer DTh, Uges DRA, Oosterhuis B and Willemse PHB (1989) A phase I and pharmacokinetic study with 21 days continuous infusion of mitoxantrone. *J Clin Oncol* **7**: 790–797

Jakobsen P, Bastholt L, Dalmark M, Pfeiffer P, Petersen D, Gjedde SB, Sandberg E, Rose C, Nielsen OS and Mouridsen HT (1991) A randomized study of epirubicin at four different dose levels in advanced breast cancer. Feasibility of myelotoxicity prediction through single blood-sample measurement. *Cancer Chemother Pharmacol* **28**: 465–469

Johnston JB and Glazer RI (1983) Cellular pharmacology of 3′-(4-morpholinyl) and 3′-(4-methoxy-1-piperidinyl) derivatives of 3′-deaminodaunorubicin in human colon carcinoma cells *in vitro*. *Cancer Res* **43**: 1606–1610

Kaye S and Merry S (1985) Tumour cell resistance to anthracyclines – a review. *Cancer Chemother Pharmacol* **14**: 96–103

Lau DHM, Lewis AD and Sikic BI (1989) Association of DNA crosslinking with potentiation of the morpholino derivative of doxorubicin by human liver microsomes. *J Natl Cancer Inst* **81**: 1034–1038

Lewis AD, Lau DHM, Durán GE, Wolf CR and Sikic BI (1992) Role of cytochrome P-450 from the human CYP3A gene family in the potentiation of morpholino doxorubicin by human liver microsomes. *Cancer Res* **52**: 4379–4384

Meijer C, Mulder NH and De Vries EGE (1990) The role of detoxifying systems in resistance of tumour cells to cisplatin and adriamycin. *Cancer Treat Rev* **17**: 389–407

Miller AB, Hoogstraten B, Staquet M and Winkler A (1981) Reporting results of cancer treatment. *Cancer* **47**: 207–214

Minow RA, Benjamin RS, Lee ET and Gottlieb JA (1977) Adriamycin cardiomyopathy – risk factors. *Cancer* **39**: 1397–1402

Mross K, Mayer U, Hamm K, Burke K and Hossfeld DK (1990) Pharmacokinetics and metabolism of iodo-doxorubicin and doxorubicin in humans. *Eur J Clin Pharmacol* **39**: 507–513

Piscitelli SC, Rodvold KA, Rushing DA and Tewksbury DA (1993) Pharmacokinetics and pharmacodynamics of doxorubicin in patients with small cell lung cancer. *Clin Pharmacol Ther* **53**: 555–561

Plosker GL and Faulds D (1993) Epirubicin: A review of its pharmacodynamic and pharmacokinetic properties, and therapeutic use in cancer chemotherapy. *Drugs* **45**: 788–856

Ripamonti M, Pezzoni G, Pesenti E, Pastori A, Farao M, Bargiotti A, Suarato A, Spreafico F and Grandi M (1992) In vivo anti-tumour activity of FCE 23762, a methoxymorpholinyl derivative of doxorubicin active on doxorubicin-resistant tumour cells. *Br J Cancer* **65**: 703–707

Robert J (1993) Epirubicin. Clinical pharmacology and dose-effect relationship. *Drugs* **45** (suppl.) 2P: 20–30

Robert J and Gianni L (1993) Pharmacokinetics and metabolism of anthracyclines. *Cancer Surv* **17**: 219–252

Schwartz HS and Kanter PM (1979) Biochemical parameters of growth inhibition of human leukemia cells by antitumour anthracycline agents. *Cancer Treat Rep* **63**: 821–825

Streeter DG, Johl JS, Gordon GR and Peters JH (1986) Uptake and retention of morpholinyl anthracyclines by adriamycin-sensitive and -resistant P388 cells. *Cancer Chemother Pharmacol* **16**: 247–252

Tidefelt U, Sundman-Engberg B and Paul C (1989) Comparison of the intracellular pharmacokinetics of doxorubicin and 4′-epi-doxorubicin in patients with acute leukemia. *Cancer Chemother Pharmacol* **24**: 225–229

Van der Graaf WTA, Mulder NH, Meijer C and De Vries EGE (1995) The role of the methoxymorpholino anthracycline (FCE 23762) and cyano-morpholino anthracycline in a sensitive small cell lung cancer cell line and its multidrug-resistant but P-glycoprotein negative and cisplatin-resistant counterparts. *Cancer Chemother. Pharmacol* **35**: 345–348

Vasey PA, Bissett D, Strolin-Benedetti M, Poggesi I, Breda M, Adams L, Wilson P, Pacciarini MA, Kaye SB and Cassidy J (1995) Phase I and pharmacokinetic study of 3′-deamino-3′-(2-methoxy-4-morpholinyl)doxorubicin (FCE 23762). *Cancer Res* **55**: 2090–2096

© Cancer Research Campaign 1998

Wagner JG (1968) Kinetics of pharmacologic response. I. Proposed relationships between response and drug concentrations in the intact animal and man. *J Theor Biol* **20**: 171–201

Wassermann K, Newman RA, Davis FM, Mullins TD and Rose KM (1988) Selective inhibition of ribosomal gene transcription by the morpholinyl anthracyclines cyanomorpholinyl- and morpholinyl anthracyclines. *Cancer Res* **48**: 4101–4106

Wassermann K, Markovits J, Jaxel C, Capranico G, Kohn KW and Pommier Y (1990) Effects of morpholinyl doxorubicins, doxorubicin, and actinomycin D on mammalian DNA topoisomerases I and II. *Mol. Pharmacology* **38**: 38–45

World Health Organization (1979) Handbook for reporting results of cancer treatment. WHO Offset Publication no. 48. Nijhoff: Den Haag, The Netherlands

Zaman GJR, Flens MJ, Van Leusden MR, De Haas M, Mulder HS, Lankelma J, Pinedo HM, Scheper RJ, Baas F and Broxterman HJ (1994) The human multidrug resistance-associated protein MRP is a plasma membrane efflux pump. *Proc Natl Acad Sci USA* **91**: 8822–8826

 *© Cancer Research Campaign 1998*